# Changing Role of PET/CT in Cancer Care With a Focus on Radiotherapy

**DOI:** 10.7759/cureus.32840

**Published:** 2022-12-22

**Authors:** Srinivasan Vijayakumar, Johnny Yang, Mary R Nittala, Alexander E Velazquez, Brandon L Huddleston, Nickhil A Rugnath, Neha Adari, Abhay K Yajurvedi, Abhinav Komanduri, Claus Chunli Yang, William N Duggar, William P Berlin, Richard Duszak, Vani Vijayakumar

**Affiliations:** 1 Radiation Oncology, University of Mississippi Medical Center, Jackson, USA; 2 Radiology, University of Mississippi Medical Center, Jackson, USA

**Keywords:** precision radionuclide therapy, cancer care, theranostics, radiotherapy (rt), positron emission tomography computed tomography

## Abstract

Positron emission tomography (PET) integrated with computed tomography (CT) has brought revolutionary changes in improving cancer care (CC) for patients. These include improved detection of previously unrecognizable disease, ability to identify oligometastatic status enabling more aggressive treatment strategies when the disease burden is lower, its use in better defining treatment targets in radiotherapy (RT), ability to monitor treatment responses early and thus improve the ability for early interventions of non-responding tumors, and as a prognosticating tool as well as outcome predicting tool. PET/CT has enabled the emergence of new concepts such as radiobiotherapy (RBT), radioimmunotherapy, theranostics, and pharmaco-radiotherapy. This is a rapidly evolving field, and this primer is to help summarize the current status and to give an impetus to developing new ideas, clinical trials, and CC outcome improvements.

## Introduction and background

Positron emission tomography (PET) integrated with computed tomography (CT) has brought revolutionary changes in improving cancer care (CC) for patients. Positron emission tomography/computed tomography (PET/CT) has become an integral part of various aspects of CC. In addition to routine use in cancer diagnosis and follow-up of monitoring the treatment results, with the evolution of personalized radiotherapy (RT), volumetric and radiobiological characteristics of individual tumors have become integrated into the RT planning (and perhaps even delivery) process. RT target volumes by anatomic imaging can be integrated into the tumor biology of PET/CT [1].

PET/CT plays a key role in treatment decision making such as palliative versus definitive treatment, choices of treatment (such as surgery *vs*. RT versus radio-chemotherapy (RCT) versus radio-biotherapy (RBT) versus* *systemic therapy alone) as well as identifying oligo-metastatic disease (OMD) status and design new ways to address those disease states or to enter patients to clinical trials. For patients planned to be treated with RT, radiation oncologists (RO) can use PET/CT to determine the gross tumor volume (GTV), clinical target volume (CTV), or nodal involvement. Working as a team with medical physicists and dosimetrists, RO will delineate the target treatment volumes after a CT simulation and image-fusion (image registration) with the diagnostic PET/CT.

PET/CT helps to plan clinical workflow for patients better compared to when only CT was available; integrated PET/CT RT workflow results in more accurate targeting of the malignant lesions now. Lesion contours can be more easily visualized and defined, reducing inaccuracies. Efficient processes for treatment planning can be achieved by reducing the registration time, fusion review time, and contouring time. Similarly, exposure of the patients to ionizing radiation in terms of integral doses can be reduced. Ultimately, imaging and RT collaborations will contribute to better planning of the course of treatment(s) increased efficiency in workflow and time management, and better patient satisfaction and CC outcomes [2] such as overall survivals, disease-free survivals, local regional controls as well as the quality of life (by decreasing acute and long-term toxicities from RT or RCT/RBT).

The advances that have already been accomplished include (a) an improvement in cancer staging, (b) identification of OMD status (and in turn causing Will Rogers phenomenon [3,4] ), (c) improved target definitions in RT, (d) decreasing RT related toxicities, (e) improved ability to dose-paint in RT to match the potential density of cancer cells, (f) improved outcomes with RT in localized diseases as well as in oligometastatic stages [5], (g) ability to monitor early treatment-related responses (and in turn being able to start new interventions early when the disease burden is still low), (h) ability to predict the prognosis better than prior to the PET/CT era, and (i) finally starting the new field of theranostics/RBT-radiobiomedicine [6].

In addition, there are great potential future possibilities for the use of new nuclide agents in diagnosis and therapy, the ability for adaptive radiotherapy, especially with the use of PET plus simultaneous magnetic resonance imaging (PET/MRI), and improving RBT [7,8]. In this paper, we will expand on most of the items discussed above, although not all of them.

## Review

Methods

Search Databases

Databases such as PubMed, PubMed Central, MEDLINE, and Google Scholar were used.

Search Strategy

The above databases were screened from October 22 to November 8, 2022, with individual keywords and key term combinations including “positron emission tomography [PET],” “computerized tomography [CT],” “radiotherapy [RT],” “cancer care,” “metabolic tumor volume [MTV],” “stereotactic body radiation therapy [SBRT],” “theranostics,” and “Precision Radionuclide Therapy [PRnucT].”

Inclusion/Exclusion Criteria

Studies in the English language from 1995 to 2022 were included.

PET/CT is changing the way we provide CC

PET is a nuclear medicine imaging technique and employs small amounts of radiotracer or radiopharmaceuticals, most often ¹⁸F-fluorodeoxyglucose (¹⁸F-FDG), to evaluate internal organs and tissues based on a variety of cellular components. In contrast to nuclear medicine imaging modalities which rely heavily on physiologic processes and as another common diagnostic tool, CT employs external x-rays from varying angles to obtain images and construct computer-processed cross-sectional images, highlighting clinically pertinent features such as bones, vessels, and organs. Integrating features and benefits from both modalities, PET/CT dual-modality imaging is consistently growing in part due to its variety of utilities, particularly in RT [9]. Specifically, PET/CT has revolutionized the identification and localization of cancerous lesions (not being able to be identified before the use of PET/CT) and has provided endless medical advances in the realms of not only cancer detection, but also improving the treatment and diagnostic accuracy, prognosis indication, and disease progression and/or response [10,11]. Delineation of tumor margins is an essential first step in the diagnosis and staging of cancerous lesions, and PET/CT has been demonstrated across various studies to improve the accuracy of margin delineation when compared to other image modalities alone [12-15]. Compared to either PET or CT alone, PET/CT has also been shown to have a high sensitivity in identifying distant metastases (Table 1) [15-17].

**Table 1:** Comparison of PET-CT versus PET alone or CT alone imaging in the selected studies. PET-CT: positron emission tomography-computed tomography, PET: positron emission tomography, CT: computed tomography, DOR: diagnostic odds ratio, PLR: positive likelihood ratio, NLR: negative likelihood ratio, CI: confidence interval, n: number, %: percentage Table reproduced from Gao et al. [16] - permission was obtained from licensed content publisher Elsevier to reproduce the table

Imaging methods	Study (n)	Patients (n)	Sensitivity (95% CI)	Specificity (95% CI)	DOR (95% CI)	PLR (95% CI)	NLR (95% CI)
Integrated PET-CT *vs.* PET alone							
Integrated PET-CT	10	1058	0.95 (0.91- 0.97)	0.96 (0.94- 0.97)	447 (196- 1020)	24.4 (15.8- 38.0)	0.05 (0.03- 0.10)
PET alone	10	1058	0.85 (0.79- 0.90)	0.95 (0.91- 0.97)	114 (65- 200)	17.5 (10.0- 30.5)	0.15 (0.11- 0.22)
Integrated PET-CT *vs.* CT alone							
Integrated PET-CT	7	745	0.97 (0.90- 0.99)	0.97 (0.95- 0.98)	1058 (295- 3798)	34.8 (20.7- 58.4)	0.03 (0.01-0.1)
CT alone	7	745	0.80 (0.73- 0.85)	0.94 (0.89- 0.97)	66.4 (27.8- 158)	14.3 (7.3- 28.2)	0.22 (0.16- 0.29)

The ability to identify distant metastasis more effectively and gauge the size of a tumor using PET/CT has allowed clinicians to more effectively stage cancerous lesions and thus be able to better evaluate a patient's prognosis and devise more effective treatment plans [10,18,19]. More effective cancer staging with PET/CT can also be achieved by evaluating the extent of FDG uptake within the lesion, which varies between tumor subtypes [20-22]. PET/CT also has utilization for patients who are undergoing or who have finished treatment as a surveillance tool for treatment response and early disease recurrence [17,18,23]. Studies have demonstrated that PET/CT has both high sensitivity and specificity in the detection of tumor recurrence, especially in patients with elevated serum tumor markers who remain asymptomatic [17,18]. For treatment response evaluation PET/CT has been shown to be effective in a variety of cancer types demonstrating a reduction in FDG uptake in cancers after initiation of RT or chemotherapy or RCT combinations [24].

The use of PET/CT can indicate the cellular processes that are occurring within the lesion such as metabolism and proliferation which can have further implications for exact tumor staging, choices of treatments, or evaluation of treatment responses [11,20,15-28]. Due to the inhibition of proliferation or the induction of apoptosis being effective targets for many anti-cancer treatments, using PET with 2-deoxy-2-[flurorine-18]fluoro- D-glucose integrated with CT (¹⁸F-FDG PET/CT) as an evaluation of lesion’s cellular proliferation has shown promise in differentiating benign versus malignant masses and in assessing for appropriate treatment response [25,29-32]. PET/CT can assess the degree of tumor oxygenation which has been shown to have a significant impact on both tumor progression and treatment response [17,26,27]. Extending its utility, recent works suggest that PET/CT can impact stage migration, and apparent improvement of overall survival (Will Rogers phenomenon [3,4]), in patients undergoing RT for a variety of cancers [33-35]. PET/CT has proven to have utilized in the initial detection and diagnosis of cancer, and it is a tool that allows for effective staging, metastasis detection, and margin demarcation. Despite the many current utilizations of PET/CT, there are seemingly more opportunities for further development as well as optimizing diagnoses, treatment, and monitoring of patients with cancerous lesions.

PET/CT has changed the way RT is being applied to CC

The Introduction of How PET/CT Changed the RT Application

Before the discovery of PET, clinical RT implementation was limited to using radiolabeled isotopes to monitor disease progression amongst a small subset of slowly proliferating diseases and malignancies. The accuracy of this method was limited in its usage as analysis of isotopes was difficult due to half-life decay, physiological removal of isotopes by the body, and the interruption of normal metabolic processes by isotopes [36]. The discovery of PET allowed for a more specific and sensitive means for imaging molecular interactions and pathways within the human body. The specificity is due to a wide range of positron-emitting radionuclides, which can be used to label specific biomarkers, biochemicals, and pharmaceuticals without interrupting normal biological function. Throughout the previous 60 years, coincidence detection of positron-emitting radionuclides has transitioned from single pairs of detectors for planar imaging to current PET scanners with arrays of detector elements encasing 25 cm in axial length and using 35,000 individual detector elements [34]. Combining PET with CT or MRI allows for the delineation of both functional and anatomic localization. Because of this, since 2001 effectively all PET scanners are physically combined with an anatomical imaging device such as a CT or, from 2010, an MRI scanner [37]. The second modality provides a high spatial resolution anatomical framework that is accurately registered with the functional PET image and additionally can be used to increase the quality of the PET image. Historically, the development of PET/CT usage within the clinic occurred in the mid-1990s, around the time when the concept of PET/MRI preclinical applications was being researched [38]. Consequently, clinical PET/CT transitioned into preclinical PET/MRI usage while, afterward, preclinical PET/MRI usage became a reality in the clinical domain. The increasing availability of hybrid PET/MRI systems has led to a scale of novel publications and opportunities for the implementation of PET/MRI in CC. While PET/CT has been an invaluable and more traditional tool for oncologic staging, there are multiple theoretical and practical advantages the emerging PET/MRI system would have over PET/CT such as in head and neck imaging [38].

PET/CT Highlighting Metabolic Tumor Volume and Its Applicationinf Stereotactic Body Radiation Therapy

Diagnostic staging and radiation planning with PET/CT have been well established along with standardized uptake value (SUV) which is a semiquantitative assessment of a tumor’s radioactivity. The maximum SUV (SUV_max_), which measures the highest intensity of uptake, serves as a useful prognostic tool but poorly represents total tumor uptake. However, metabolic tumor volume (MTV), along with total lesion glycolysis (TLG), can assess the metabolic activity of tumors and volumetric burden with potential prognostic capabilities. The limitations of SUV_max_ combined with the ability to assess the total metabolic activity of MTV create an ideal scenario to employ MTV. Moreover, a meta-analysis has indicated that MTV can serve as a superior prognostic tool when compared to SUV_max_, specifically for patients with head and neck cancer [39]. Similarly observed with the application of PET/CT to measure immunotherapy response in patients with metastatic melanoma, MTV, along with peak SUV (SUV_peak_) and TLG, can serve as promising predictors for the final response after therapy [40]. Additionally, the predictive recurrence value of MTV can aid in clinical decision-making for patients, particularly with stage IIB-IVA cervical cancer [41]. Regarded as a metastasis-directed therapy with key features of a non-invasive nature and short treatment duration, stereotactic body radiation therapy (SBRT) delivers ablative radiation doses with the goals of removing tumors of low burden and yielding local control [42].

The application of SBRT with PET/CT has involved oligometastatic prostate cancer (OMPC) and non-small cell lung cancer (NSCLC) [42-46]. A meta-analysis of 356 abstracts and 10 studies concluded that SBRT effectively controls overall disease burden in OMPC patients; in this study, PET/CT staging occurred in 92.4% of all patients which suggests the significant role of PET/CT in SBRT [43]. In cases worth avoiding the start/escalation of palliative androgen deprivation therapy which remains the standard therapy for OMPC, gallium-68-prostate-specific membrane antigen (^68^Ga-PSMA) PET/CT-based SBRT is a viable alternative that further demonstrates the utility of PET/CT [44]. Additionally, PET/CT has improved SBRT treatment in patients with NSCLC by advancing the development of prognostic PET/CT radiomic signature, specifically textural features information correlation 2 and strength [46].

PET/CT Decreasing Complications During Radiation Therapy

Addressing complications by decreasing irradiation of normal tissue has been seemingly a perennial priority surrounding the use of RT/RCT, and these risks can be modeled with dose and volume-treated approaches [47]. Beginning in the 1980s, the response of normal tissues to RT led to increased research and development of mathematical models for radiobiological responses such as normal tissue complication probability (NTCP) which can predict radiation-induced morbidities. Additionally, the development of advanced, modern technology has enabled the introduction of the use of RT to previously difficult-to-treat tumors (target volumes close to organs at risk and pediatric tumors). As a result of reducing the volume of normal tissues exposed to RT, therapies can decrease NTCP [48].

By the same token, PET/CT-guided RT has been reviewed for its high sensitivity and specificity in identifying tumor sites to minimize unnecessary radiation as well as geographical misses. Given that errors in delineation methods result in either suboptimal treatment or increased normal tissue irradiation, an ability to improve delineation, such as those found with PET/CT, will decrease normal tissue damage [12]. In the treatment of cancers in areas that require significant sparing of adjacent normal tissues such as those in the head and neck, accurate target delineation becomes paramount for optimal treatment [49,50]. For patients with meningiomas, gallium dotatate PET/CT confirmed diagnoses and delineated target volumes in the osseous structures and falx; treatment with PET/CT decreased tumor activity three months post-RT whereas MRI alone did not [49]. Similarly, and with high accuracy sensitivity, and specificity, PET/CT demonstrated an ability to diagnose nasopharyngeal carcinoma in cases with malignancies undetectable by MRI which led to no relapses after a median of 48.3 months (about four years) [49].

Disease site-specific examples of the advances made with the use of PET/CT

Brain

For patients diagnosed with brain tumors, the use of PET/CT has increased significantly over the last several years with four key areas of investigation emerging: tumor margin delineations, response to systemic therapies, pseudo-progression versus radio-necrosis versus progression, and response to immunotherapy through t-cell imaging [9].

Compared to conventional modalities such as MRI alone or CT alone, PET/CT tracers (such as ^18^F-FDG,^18^F-DOPA, and ^18^F-FET) were employed to target tumor margin delineations yields a clearer image of the brain tumor edges, consequently improving effective resection by reducing the risk of complications. Some complications include iatrogenic strokes, meningitis, and hemorrhage/hematoma. Among many options in the use of systemic therapy in combination with RT include the need to cross the blood-brain-barrier (BBB) to act on cancer cells nested in the brain, and examples include chemotherapy, hormonal therapy, targeted drugs, and immunotherapy RBT. PET/CT allows for better targeting of treatment to minimize side effects and optimize post-treatment recovery. Commonly seen after RT, new enlarging areas emerge in a process called pseudo-progression [51]. These new masses often lead to premature discontinuation of treatment and risk future complications. PET/CT incorporates hybrid imaging as opposed to standard morphological imaging which has been shown to significantly influence initial staging and patient management [52]. The term radio-necrosis refers to the unintended destruction of healthy tissue in the body collateral with the destruction of malignant tissue during radiation therapies. T-cell imaging can assist in understanding the body’s response to immunotherapy [53]. T-cell-specific PET/CT imaging strives to provide clear visualizations of tumor-infiltrating lymphocytes, which are responsible for recognizing and terminating the cancer cells.

Despite the lack of current routine standardizations of PET/CT, its variable utility and opportunities for future investigations constitute the need to further research- laboratory, preclinical, translational and clinical that will lead to improved outcomes in brain tumors in the next five to ten years.

Head and Neck

**Figure 1:**
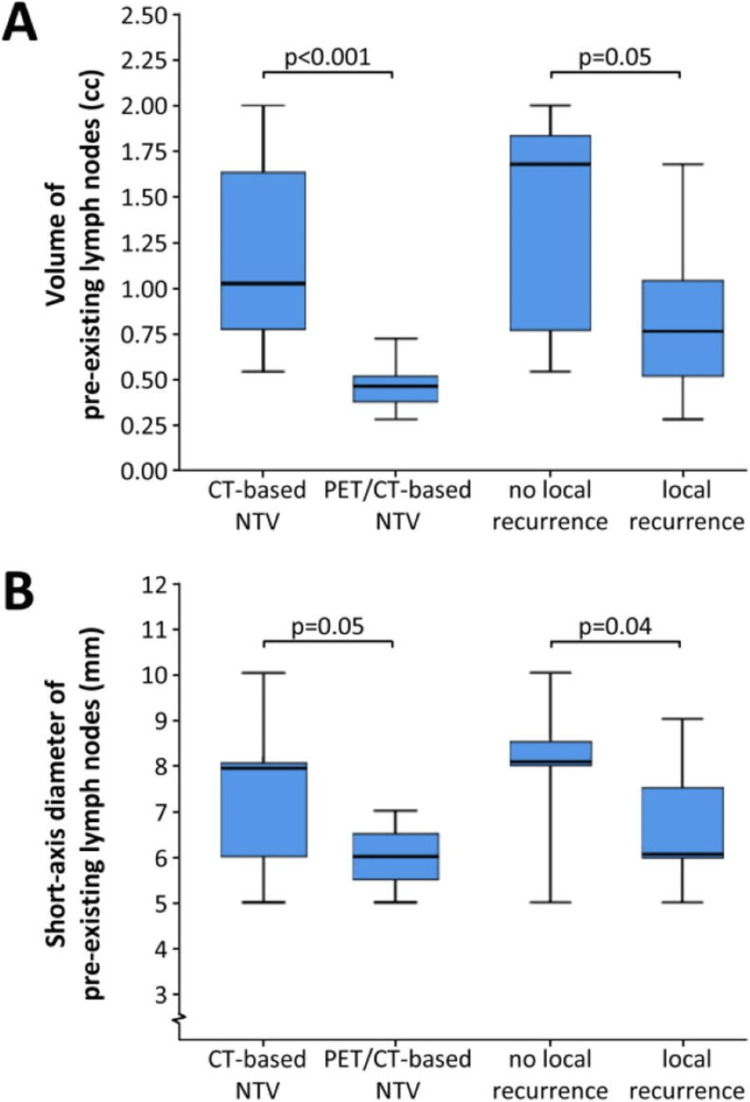
CT-based versus PET/CT based normal tissue volumes comparison PET/CT: positron emission tomography/computed tomography, PET: positron emission tomography, CT: computed tomography, NTV: nodal target volume definition This image is reproduced from Van De Bosch et al. [13] - available via Creative Commons CC-BY-NC-ND license

Metastases from head and neck cancers although rare do occur in lungs, mediastinal lymph nodes, liver, and bone, presents dilemmas to clinicians on treatment plans and limited improvement in outcomes. However, the usage of ^18^F-FDG-PET/CT is widely accepted in current pre- and post- treatment of head and neck patients [54]. The usage of whole-body metabolic functional imaging by ^18^F-FDG PET along with anatomical framing by CT allows for the identification of distant metastasis of cancers within (for example, lymph nodal improvement or bone erosions) or outside to the head and neck regions. Detection of nodal metastases by traditional mechanisms such as CT and MRI is dependent mainly on nonspecific anatomical characteristics with reduced capability to discriminate between metastatic and non-metastatic lymph nodes of small size. ^18^F-FDG-PET/CT is able to accurately delineate metastases allowing for more effective tumor staging and grading. Molecular imaging using ^18^F-FDG-PET/CT provides a non-invasive and quantitative assessment concerning the functional process of glucose metabolism. Because an increased glucose metabolism is characteristic of squamous cell carcinoma, a common cancer of the head and neck, higher accuracy of ^18^F-FDG-PET/CT in the detection of nodal metastases of head and neck cancers was demonstrated compared to traditional methods [13]. The capability of ^18^F-FDG-PET/CT to indicate metabolic processes, such as glucose metabolism, is the main contributing factor of its superiority over conventional imaging. Previous head and neck cancer studies compared the two modalities (CT *vs.* PET/CT) using the GTV as comparators. GTV was determined using clinical examination and, diagnostic anatomic imaging using characteristics indicative of macroscopic disease involvement. Notable GTV responses to ^18^F-FDG-PET/CT directed target volumes- based RT/RCT/RBT are indicated by such target volume definitions improving regional control and survival, a reduction in isolated recurrences in the electively treated neck cancers, target volume transformations, and the guidance of gradient dose prescription with a de-escalation of the elective dose (Figures 1A, 1B).

The results support the concept of target volume transformation and demonstrate the potential of ^18^F-FDG-PET/CT to guide RT dose de-escalation in elective neck treatment in head and neck cancers. Not only does ^18^F-FDG-PET/CT assist in staging and RT planning but also plays a central role in the assessment of response to treatment in subsequent monitoring [13]. More investigations are needed to fully understand just how beneficial ^18^F-FDG-PET/CT treatment could potentially be for this body site.

Breast

**Figure 2:**
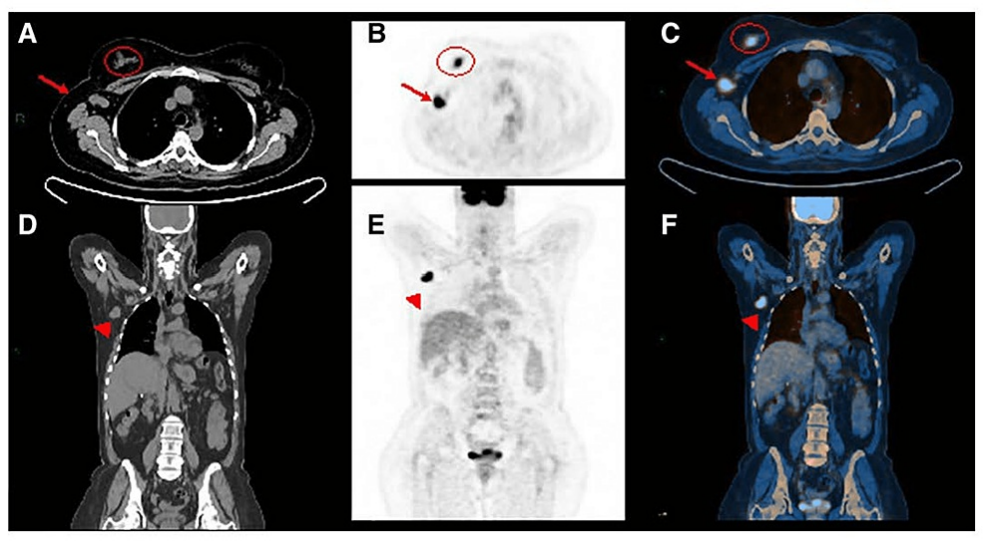
CT - based versus PET/CT based comparison of invasive ductal cancer of the right breast CT of the whole body: Axial (A) and coronal (D) views show enlarged node in the right axilla (A, arrow; C) associated with an irregular mass within the right breast (A, circle). PET of the whole body: Axial (B) and coronal (E) views demonstrate high flurodeoxyglucose uptake for both the mass and the enlarged node in the axilla that is also confirmed by hybrid imaging PET/CT (C, F) CT: computed tomography, PET: positron emission tomography, PET/CT: positron emission tomography/computed tomography This image is reproduced from Marino et al. [56] - available via Creative Commons CC-BY-NC-ND license

Increasingly sensitive imaging modalities allow for specificity, diagnostic accuracy, precise detection of tumor normal tissues margins, refined image quality, and overall improved modeling of target delineation of RT treatment for breast cancer [55]. Generally, breast cancer metastases to axillary lymph nodes more frequently than other areas, a major prognostic factor in breast cancer. The usage of FDG-PET/CT is helpful for the detection of positive lymph nodes. Given RT techniques require target delineations, PET/CT co-registration using deformable methods is advantageous when planning RT treatment in breast cancer patients. The involvement of PET/CT optimizes RT treatment planning for breast cancer patients and allows clinicians to accurately analyze anatomic patterns of tumor metastasis and breast cancer recurrence and therefore adapt RT treatments accordingly. Through previous studies, FDG-PET/CT involvement has led to substantial clinical impacts in high-risk primary breast cancer with a diagnosis from accurate detection of distant metastases and relevant incidental findings including diagnosis of synchronous diseases [33] as well as OMD. Furthermore, the RTOG atlas was found to be insufficient in adequately covering regional nodal areas such as positive lymph nodes after neoadjuvant systemic chemotherapy [10]. Given the lack of RT contouring guidelines for patients undergoing neoadjuvant systemic therapy, pre-systemic therapy- PET/CT fusion images are beneficial in contouring sites of positive lymph nodes based on the lymph node levels (axillary, internal mammary, supraclavicular) and ensuring they are in the CTV. Usage of image registration with PET/CT has displayed nodal recurrences and significant sites of nodal metastases that were outside of Radiation Therapy Oncology Group (RTOG) and European Society Radiation Oncology (ESTRO) contouring guidelines. External beam RT, the most common RT for patients with breast cancer poses risks of toxicities including lymphedema, late secondary malignancies and endangers other organs including the heart, lungs, and brachial plexus, especially when level III axillary nodes and internal mammary lymph nodes need to be included in the target volumes [55]. Utility of PET/CT is valuable for increasing efficacy of external beam RT treatment planning through accurate displays of localized lymph node metastasis and delineation of malignant tissue, thus improving precision of high dose delivery and irradiated volumes. Given the promising benefits in helping define the GTV, CTV, and MTV, PET/CT use should be considered in every case of lymph nodal RT in breast cancer for improving treatment planning and overall improvements in treatment outcomes (Figures 2A-2F) [56].

Pediatrics

Within the past twenty years, the use of PET/CT has grown in areas of imaging and research for pediatric care [57-61]. Νon-invasive PET/CT has enabled a change toward clinical staging rather than previously employed surgical staging, such as staging laparotomies and has also paved the path to better modify the course of treatment based on the specific tumor responses [58]. Employing FDG-PET/CT for direct evaluation of tumor growth has greatly enabled clinicians to distinguish viable, residual, or recurrent tumors from necrosis as well as post-therapeutic changes [59]. The addition of CT transmission scanning has shortened total acquisition time-the duration necessary to record the signal-thus being particularly advantageous in the evaluation of pediatric cancers [60]. Furthermore, utilizing the dual-modality PET/CT imaging systems offer in-depth diagnostic capabilities, which is especially necessary in pediatric cases. While performing a pediatric PET/CT scan, increased attention needs to be paid in limiting the patient’s exposure to the ionizing radiation dose. Moreover, minimizing the scan duration with the addition of sedation times prioritizes the goal of overall patient experience [61].

**Figure 3:**
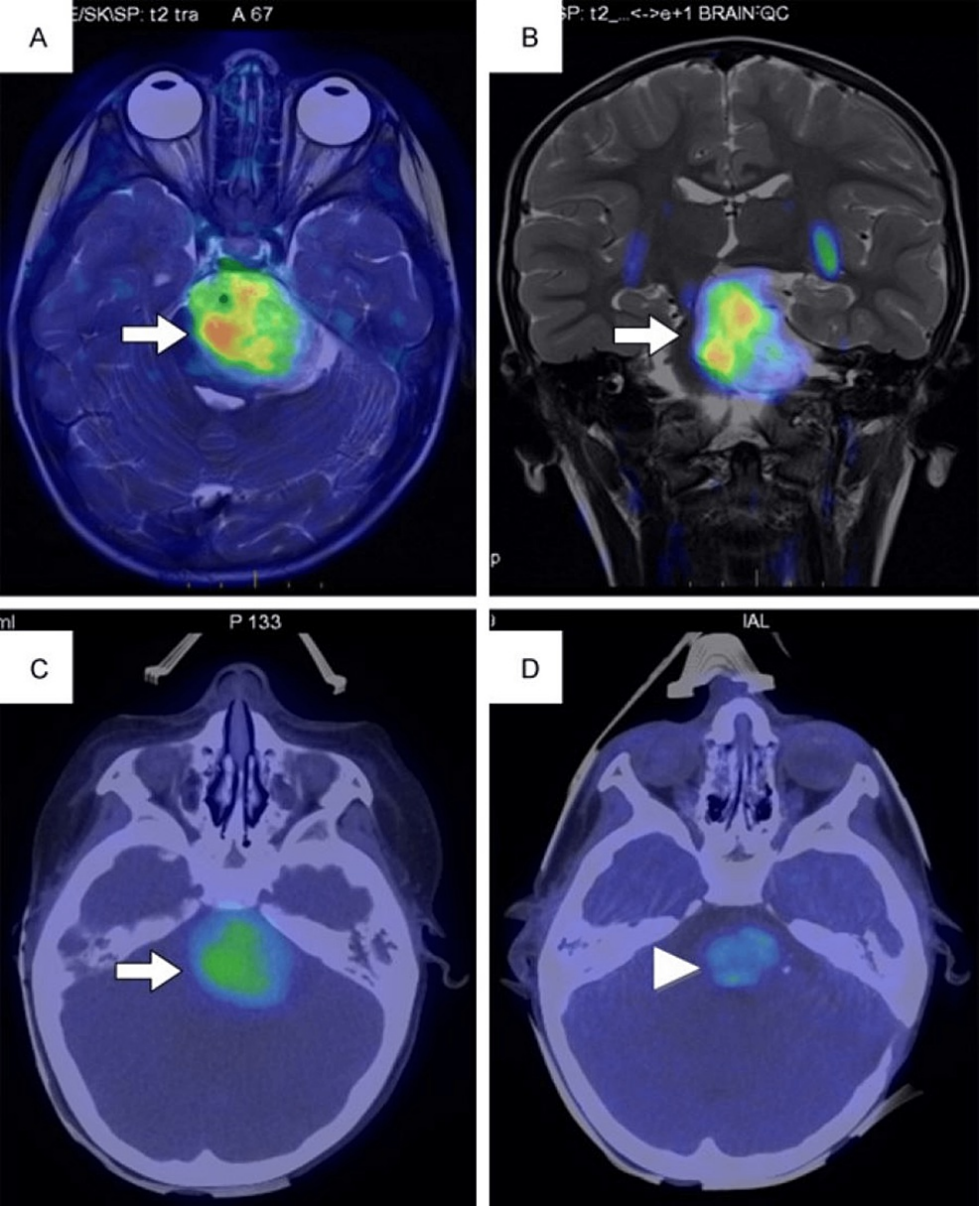
18F-DOPA scan of neuroendocrine embryonal tumor before and after therapy documenting treatment response ^18^F-DOPA: Fluorine-18-I-Dihyroxyphenylalanine Axial (A) and coronal (B) magnetic resonance imaging - positron emission tomography fusion images and axial positron emission tomography/ computed tomography (C) images showing brainstem uptake (arrow); axial positron emission tomography/computed tomography (D) after systemic chemotherapy and proton therapy shows lower brainstem uptake (arrowhead) This scan image is reproduced from Masselli et al. [62] - permission obtained from e-Century Publishing Corporation

A recent review has indicated the use of PET/CT for malignancies such as brain tumors (most common), osteosarcoma, ewing sarcoma, neuroblastoma, and lymphomas [62]. In addition to its broad use, numerous radiotracers like FDG, ^11^C- Methionine (^11^C-MET), Fluorine-18-I-Dihyroxyphenylalanine (^18^F-DOPA), Fluorine-18-fluro-ethyl-tyrosine positron emission tomography (^18^F-FET PET) further diversify the application of PET/CT, specifically for brain tumors [59]. Depending on the neoplasm, the optimal radiotracer employed with PET/CT can vary; for example, ^18^F-DOPA has been proven to show superior contrast when compared to F-FDG and is depicted for a pediatric case in Figures 3A-3D [62].

However, compared to PET/CT use in adults, clinical assessment and decisions for PET/CT use in pediatric cases are complicated more by the impact of radiation exposure for diagnosis, staging, and follow-up in children whose organs and tissues are more radiosensitive [62]. It is worth mentioning that this caveat is suggested to serve as a consideration for clinicians when deciding on a diagnostic tool rather than serve as a limitation. Standard clinical applications of PET/CT for pediatric cancers are still in the preliminary stages, but the described benefits of PET/CT yield significant interest for further investigations.

Lymphoma

The use of PET scans in the staging of lymphomas has emerged from a minor use to one of critical value. The introduction of PET/CT in the management of lymphomas allows for accurate and reproducible staging, providing a pathway for consistent beneficial outcomes. This can be seen in medicine’s abandonment of the Ann Arbor classification and adopting of the 2014 Lugano classification [63]. This transition recognized the much-improved diagnostic accuracy and efficacy of PET/CT for the routine staging of all lymphomas compared to stand-alone CT staging, abandoning the use of B symptoms (weight loss, night sweats, fever) as the critical diagnosable signs of disseminated disease in non-Hodgkin’s lymphoma.

**Figure 4:**
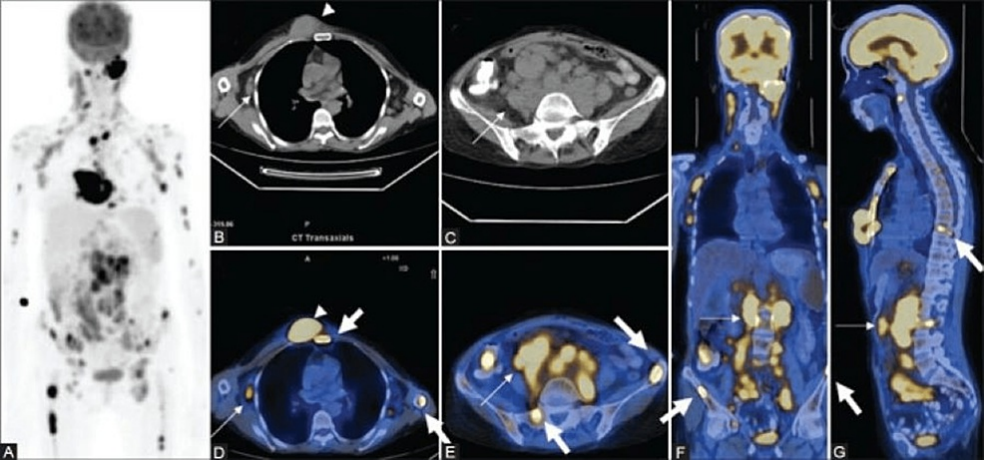
Use of PET/CT in the staging of lymphomas including the ability to diagnose bone marrow involvement Maximal intensity projection image (A) in a patient of diffuse large B-cell lymphoma shows extensive sites of involvement visualized as areas of increased fluorodeoxyglucose uptake. Trans-axial computed tomography images (BB, C show axillary and abdominal lymphadenopathy (thin arrows) ad a large subcutaneous nodule (arrowhead). Fused PET/CT images (axial D, E; coronal F; and sagittal G) show sites of bone marrow involvement (thick arrows) in the sternum, spine, and iliac crest, over and above the lesions picked up on CT This image is reproduced from D'souza et al. [64] - available via Creative Commons Attribution - Noncommercial- Share Alike 3.0 Unported license

The use of PET/CT has also introduced the consideration of previously omitted criteria in the classification and staging of lymphomas and tissue metabolism. The introduction of tissue metabolism in the staging of lymphomas allows physicians to obtain a more inclusive and complete assessment of the lymphoma, providing a direct pathway for assessing efficacious treatment options. FDG-PET/CT is the cornerstone of Hodgkin and non-Hodgkin’s lymphoma staging, restaging, prognostication, monitoring therapy, and detecting recurrence. The advantage of using FDG-PET/CT over conventional CT imaging is the ability to detect metabolic changes within the lymphoma and surrounding tissues before it becomes clinically visible as shown in Figures 4A-4G [64].

This allows physicians to detect and treat the existing lymphoma, providing more advantageous prognoses and results. This multi-faceted approach to the assessment of lymphomas has allowed physicians to gain insight into the intricacies of lymphomas and their characteristics, providing greater possible avenues for therapeutic techniques. The use of PET/CT has greatly improved the efficacy of lymphoma prognosis as well as the efficacy of the proposed treatment plan. In two prospective studies involving patients with Hodgkins lymphoma, the integration of PET/CT altered the proposed treatment plan for 8.1% of patients in one study and 10% in the other [65]. Overall, the use of PET/CT allowed for the upstaging of 18% of the patient’s lymphoma to an advanced staging, providing a more efficient treatment outlook and management [66]. The change in treatment plan was the result of the more accurate and precise description of the lymphoma with PET/CT versus one of CT alone. When comparing one-year mortality rate of patients with aggressive non-Hodgkin lymphoma, the mortality of patients with completion of first-line therapy regimen decreased with the use of PET/CT versus CT alone. This suggests that adult non-Hodgkin lymphoma patients with PET-directed management results in better patient outcomes. The use of PET/CT in patients with Hodgkins and non-Hodgkins lymphoma improves patient description and overall patient survival.

An overview of all cancer sites and the future

Table 2 shows the current state of the art utility of PET/CT in CC with a focus on diagnostic, prognostic and treatment-monitoring perspectives for selected disease sites based on the above discussions.

**Table 2:** PET/CT’s current and future role in diagnostic related applications – this paper’s authors’ point of view PET/CT: positron emission tomography/computed tomography

Disease site	Standard of practice already?	Evolving? promising in the future	Role in oligometastatic detection	Role in prognosticating and treatment response monitoring?
Brain	No	Yes	No	May be- not routine yet
Head and neck	Yes	Yes	Yes	Yes
Lung	Yes	Yes	Yes	Yes
Esophagus	Yes	Yes	Yes	Yes
Lymphomas	Yes	Yes	No	Yes
Prostate	Only in recurrent	Yes	Yes	May be- not routine yet
Cervix	Yes	Yes	Yes	Yes
Breast	Yes	Yes	Yes	May be-not routine yet
Pediatric tumors	Depends on the disease site	Yes	Yes, still evolving though	Yes, and depends on the disease site

Table 3 shows the current and future potentials of PET/CT and PET/MRI with a focus on treatment and theranostics.

**Table 3:** PET/CT’s current and future role in therapeutic related applications – this paper’s authors’ point of view PET/CT: positron emission tomography/computed tomography

Disease site	Standard of practice, already?	Evolving? promising in the future	Role in oligometastatic detection	Role in prognosticating and treatment response monitoring?
Brain	No	Yes	No; yes, in the future	Yes, In the future
Head and neck	No	Yes	No; yes, in the future	Yes, in the future
Lung	No	Yes	No; yes, in the future	Yes, in the future
Esophagus	No	Yes	No; yes, in the future	Yes, in the future
Lymphomas	Yes, in selected circumstances	Yes	No; yes, in the future	Yes, in the future
Prostate	Yes, in selected cases of castrate resistant status	Yes	Yes, in selected cases of castrate resistant status	Yes, in the future
Cervix	No	Yes	No; yes, in the future	Yes, in the future
Breast	No	Yes	No; yes, in the future	Yes, in the future
Pediatrics	Yes, in some diseases	Yes, but the integral dose can be of concern	Yes, in some disease circumstances	Yes, in the future, with attention need to be placed on the integral dose

Tables 2, 3 are conceptualized by the authors as a work in progress and are likely to change rapidly in the coming years. The tables are self-explanatory and no further discussion on them will be further entertained.

Case studies as examples for role of PET/CT in radiobiotherapy

**Figure 5:**
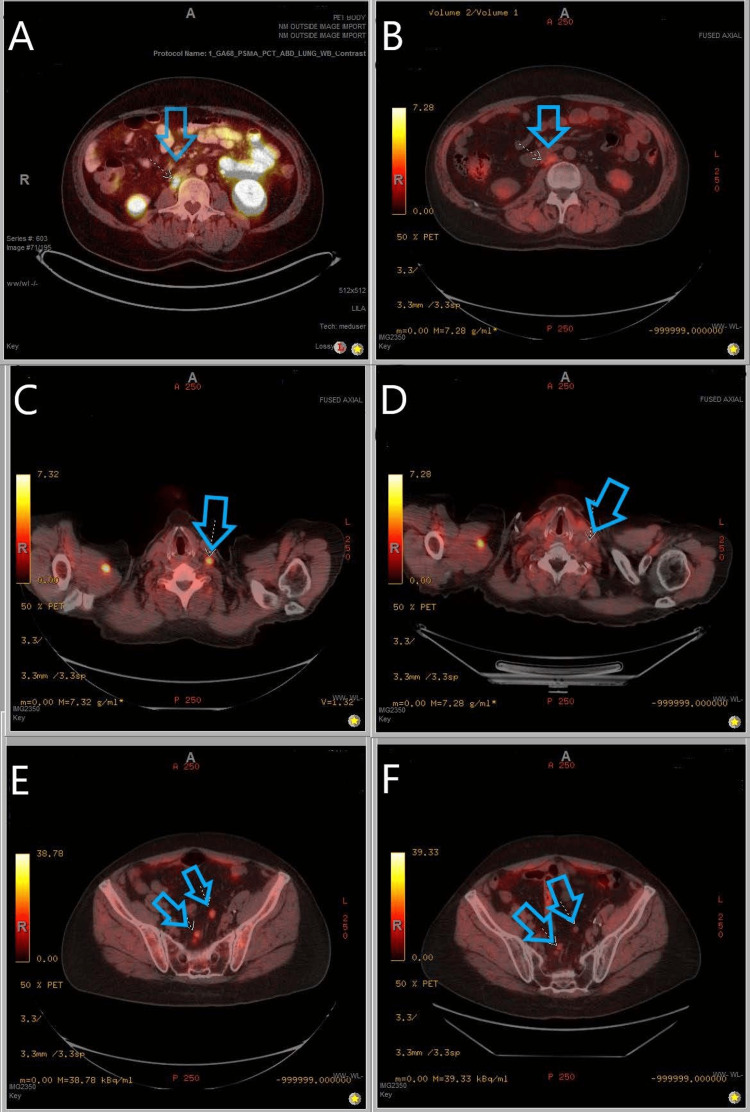
Demonstration of PET-directed prostate cancer oligometastatic disease treated with radiation therapy (A) Avid abdominal node prior to RT* *(B) Same abdominal node showing less avidity post-RT (C) Avid neck node prior to RT (D) Same neck node post-RT (E) Avid pelvic nodes prior to RT (F) Same pelvic nodes post-RT PET: positron emission tomography, RT: radiation therapy This image is permitted through the signed consent from respective University of Mississippi Medical Center patient

**Figure 6:**
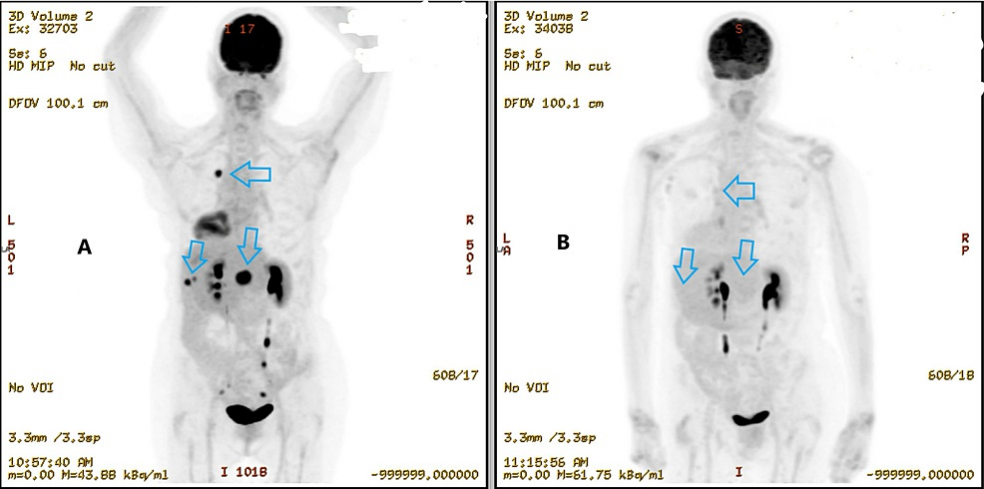
Potential abscopal effect realized by follow-up PET demonstrating global reduction in PET-avidity after immunotherapy-boosted, hypo-fractionated RT to only the central liver lesion and intramammary node (A) Prior to RT and immunotherapy* *(B) Post-RT and immunotherapy PET: positron emission tomography, RT: radiation therapy This image is permitted through the signed consent from respective University of Mississippi Medical Center patient

**Figure 7:**
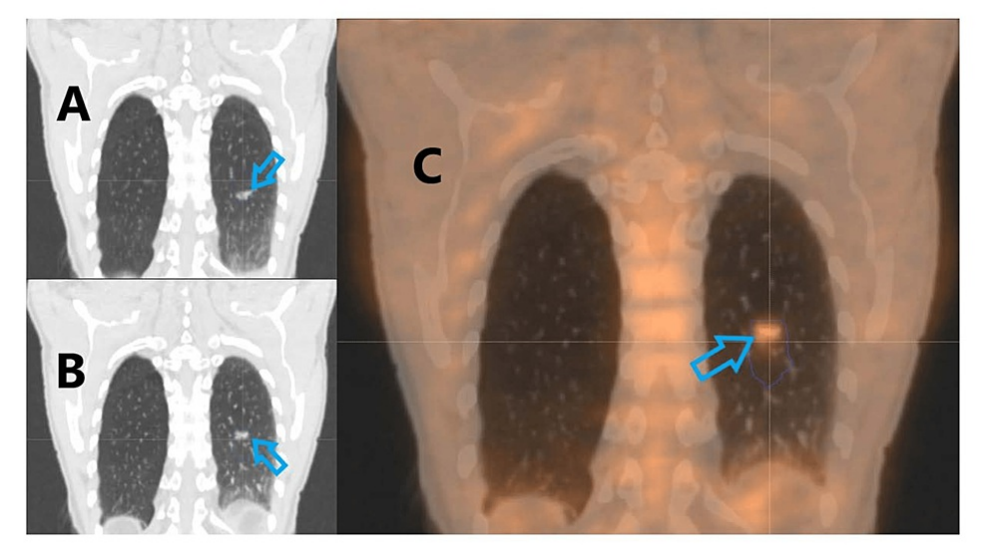
Lung tumor realized by 4DCT showing superior and inferior extremes of motion (A) Inferior extremes of motion (B) Superior extremes of motion (C) Demonstration of PET/CT's potential not only for tumor identification, but also for tumor real-time tracking. 4DCT: Four-dimensional computed tomography, PET/CT: positron emission tomography/ computed tomography This image is permitted through the signed consent from respective University of Mississippi Medical Center patient

The RBT is defined as combinations of systemic interventions with RT as a force multiplier [4]. There synergistic combinations emerging rapidly are leading to improved outcomes, and potential dose-de-escalation of radiotherapy and systemic therapy. The mechanisms of such synergistic success of RBT include immune-modulation, immune-stimulations, neoantigen[s] release, abscopal effect, spatially focused RT to PET/CT detected lesions and OMD management [67-69]. The case examples from the authors institution, University of Mississippi Medical Center (Figures 5-7) given here are illustrative of the successes of these new interventions using the concept of RBT [70-72].

 

Precision radionuclide therapy and theranostics

Theranostics is an evolving new concept in cancer therapy. It is used to describe the combination of using one radioactive drug to identify and a second often related radioactive drug to deliver therapy to treat the tumor and any metastases. In addition, targeted radionuclides are being used as imaging agents to select patients for therapeutic applications known as theranostics.

Precision Radionuclide Therapy (PRnucT)

PRnucT can be highly effective in CC with an aim to cure or in alleviating symptoms in patients with untreatable advanced-stage cancers needing palliation. Traditional radiation therapies use externally or internally delivered x-rays, protons, or other high-energy particles to target and destroy cancer cells. While improved technological approaches have reduced healthy tissue related complications, traditional RT still causes side effects that can be difficult for some patients to tolerate. The current novel approach to tumor treatment PRnucT uses radiation treatments in precision oncology and delivers a new class of cancer therapies. The goals of PRnucT development are to combine alpha-, beta- or gamma-particle emitting radionuclides with peptides, antibodies, or small molecules, and to develop therapies with high specificity for certain types of tumors. Delivered to the patient intravenously, PRnucT is designed to deliver therapeutic radiation doses with high precision directly to the cancer microenvironment and minimize radiation exposure to normal tissues.

Theranostics

Radionuclides can be delivered using precision targeting agents to image a patient for cancer followed by targeted radionuclide therapy. One gamma radionuclide is used to image tumors while an alpha/beta labeled radionuclide to deliver radiation directly to target tumors is known as theranostics. Gallium-68 is a commonly used radionuclide in diagnostic imaging tools a single highly effective targeting molecule and an imaging agent. Theranostic radionuclide imaging agents target the same cells and tissues as the accompanying therapeutic agents like Lutetium 177.

Theranostics allows oncologists to image treatable cancer cells accurately, select patients for appropriate therapeutic applications, watch precisely where therapeutic agents will be delivered, and monitor the ability of the therapeutic agent to the favorable or unfavorable response of tumors over time.

Finally, theranostics give oncologists confidence with these new and highly effective agents and provide precision treatment plans for their cancer patients. Targeted radionuclide therapies have the potential to deliver therapeutic doses of radiation with high specificity to certain types of tumors. Replacing a therapeutic radionuclide with an imaging-specific radionuclide allows the clinician to see where the therapeutic agent will be delivered and this can help dose paint the cancer in combination with other forms of RT.

Emerging use of PET/CT: biology-guided radiation therapy

In a few limited clinics, PET is not only being used to guide RT target volumes, but also RT delivery. PET signal from gross tumors is visualized real-time on a new linear accelerator fitted with PET detection allowing not only for confidence in target localization, but also the ability to adapt online to actual tumor position rather than relying only on pre-treatment imaging or online imaging that is without this biological information. Truly, PET’s known value to clinical outcomes is only the tip of the iceberg, with the excitement on the horizon [73].

## Conclusions

This paper needs to be considered only as a primer and a work in progress. There are potentials in this field in terms of improving CC that can be accomplished if investments are made in the following areas in terms of research and development: building multidisciplinary teams consisting of nuclear medicine experts, radiation oncologists, medical physicists, radiopharmaceuticals experts, medical oncologists, and clinical trials staff; conducting phase I, II and III clinical trials, educating the next generation of health care workers who are well trained in the arts and sciences of these disciplines, developing investigator-initiated clinical trials of phase I and II types in individual institutions and taking them to the cooperative group settings for phase III studies, and funding investments from National Institutes of Health and National Cancer Institute in the United States and by other equivalent institutions in other countries and pharmaceutical industry.

The field of nuclear imaging and molecular medicine is rapidly reemerging. Without a good knowledge of the changing paradigms in this important discipline, students and current practitioners of CC will not be able to provide optimal care to our patients. This communication is an attempt to summarize the current status with case examples and provide a quick review of the current state of the art of the field.
